# Coumarin-modified cellulose as an efficient adsorbent for cationic dye removal from aqueous environments: synthesis, characterization, and adsorption performance

**DOI:** 10.1038/s41598-025-17789-2

**Published:** 2025-09-12

**Authors:** Heba E. Saad, Ahmed Mahmoud

**Affiliations:** Department of Chemistry, Faculty of Science, Mansoura University, Mansoura, 35516 Egypt

**Keywords:** Modified cellulose, Coumarin-thiazole, Brunauer–Emmett–Teller, Rhodamine b dye and real wastewater samples, Chemistry, Analytical chemistry

## Abstract

In this study, a novel cellulose-based adsorbent was developed through a two-step chemical modification process involving commercially available cellulose, sodium periodate as an oxidizing agent, and a coumarin-thiazole derivative as the functionalizing agent. The modified cellulose was successfully prepared and characterized using FTIR analysis confirmed the formation of C = N stretching vibrations with a new peak at 1729 cm⁻¹. FESEM images showed a rougher and more irregular in texture and the EDX confirmed nitrogen and sulfur peaks corroborates the presence of the coumarin-thiazole compound on the cellulosic fiber, but BET analysis determined that COMC exhibited a surface area of 7.933 m²/g, a total pore volume of 0.05976 cm³/g, and an average pore diameter of 25.207 nm. The performance of the modified cellulose was assessed for its efficiency in adsorbing and separating cationic dyes. The resulting material exhibited significant adsorption capabilities, with maximum capacities reaching 142.24 mg/g for methylene blue (MB) and 68.49 mg/g for rhodamine B (RhB). To gain insights into the adsorption behaviour, several operational parameters were systematically investigated, including pH, initial dye concentration, contact time, temperature, and adsorbent dosage. An optimal adsorbent mass of 0.05 g was identified for the effective removal of 80 mg/L MB and 25 mg/L RhB. Adsorption equilibrium data conformed closely to the Langmuir isotherm model (R^2^ > 0.985) and followed the pseudo-second-order kinetic model, suggesting monolayer adsorption and chemisorption mechanisms. Thermodynamic analyses indicated that the dye adsorption was both spontaneous and exothermic, as evidenced by negative Gibbs free energy (ΔG^o^) and enthalpy (ΔH^o^) values. Furthermore, the modified cellulose demonstrated strong applicability in treating real wastewater samples, achieving dye removal efficiencies exceeding 91%. The inherent functional versatility of regenerated cellulose thus presents a promising strategy for the efficient removal of a wide array of cationic dyes from aqueous environments.

## Introduction

Among the pollutants of concern are dyes aromatic organic compounds widely utilized in industries such as textiles, pharmaceuticals, leather processing, and cosmetics. Their xenobiotic characteristics, high chemical stability, and reactive behaviour render conventional wastewater treatment systems largely ineffective in fully removing them^1,2^. Consequently, a broad spectrum of advanced treatment technologies has been explored for dye-contaminated effluents. These include microbial degradation under both aerobic and anaerobic conditions^3,4^, advanced oxidation techniques^5–7^, reverse osmosis^8^ physico-chemical approaches^9^, electrochemical methods^10^, coagulation processes^11^, biological and enzymatic treatments^12^, membranes filtration^5^ and adsorption-based techniques^13,14^.

Adsorption may prove to be the cheapest and simplest among the rest. Unlike some other methods, adsorption could apply to both inorganic and organic contaminant removal processes in nearly minimal space and time^15^. As a definition, adsorption is the process a s called the attachment of species regarded as contaminants to the surface of a solid porous matrix^16^. For adsorption from aqueous solution, the properties of the particular adsorbents are very important with respect to the adsorption capability. Moreover, because of the variation of the charges, the different pollutants cannot be efficiently removed using a single adsorbent material. From this perspective, there is an urgent need for adsorbents with high selectivity, high adsorption capacity, and low cost^17^. One of the most important goals in the design of an adsorbent is to maximize the adsorption sites per mass of material to improve the adsorption capacity and lower the cost of the process. Previous studies have described the development of porous cellulose-based bio-adsorbents, extensively functionalized with carboxyl and amino groups through graft copolymerization techniques^18,19^. These modified cellulose structures exhibited well-defined porosity and a high density of active binding sites, which collectively enhanced mass transfer efficiency and enabled remarkably high adsorption capacities for various dyes and metal ions from aqueous media^20–22^.

Synthetic dyes are increasingly being ingested into a variety of industries. The worst offenders are the textile and dyestuff industries, which both use enormous amounts of water and pollute its reservoirs with a staggering amount of dyestuffs. This effluent water has, for a long time, been considered an important ecological challenge because it results in the pollution of the environment and natural resources and poses a threat to human life^23–25^. Ecological disturbance occurs through the pollution of water sources with various dyes; one very popular branded cationic dye used in the textile industry is Rhodamine B dye (RhB) and Methylene Blue (MB). Rhodamine B (RhB) is a synthetically manufactured xanthene-based cationic dye, widely utilized in paper printing as well as in textile and food industries for its colouring properties^26,27^. Exposure to RhB poses significant health risks to both humans and animals, potentially causing skin, eye, and respiratory irritation upon ingestion^28^. Moreover, clinical evidence has confirmed that RhB contaminated drinking water exhibits carcinogenic, neurotoxic, and long-term harmful effects^29^. Therefore, RhB-containing wastewater requires meticulous treatment prior to discharge into natural water systems^30^. Methylene Blue (MB), another prevalent pollutant, is similarly categorized as highly toxic and potentially carcinogenic^24,31^. Due to its structurally stable combination of aromatic and aliphatic functional groups^32^, MB demonstrates strong resistance to biodegradation processes^33^.

In recent years, various organic compounds including 8-hydroxyquinoline, carboxylic acids, β-cyclodextrin, and polyvinylamine have been chemically grafted onto cellulose substrates to enhance their effectiveness in pollutant removal^34–36^. Despite these advancements, achieving selective adsorption and efficient separation of diverse contaminants continues to present significant challenges. In this context, the present study investigates the adsorption performance of a newly engineered cellulose-based material functionalized with a coumarin-thiazole moiety, specifically targeting the removal of methylene blue (MB) and rhodamine B (RhB) dyes from aqueous media. In order to enhance the efficiency of dye removal, several experimental parameters including the initial dye concentration, pH of the solution, amount of adsorbent, and contact duration were methodically adjusted. Additionally, comprehensive studies of adsorption kinetics and thermodynamics were carried out to better understand the equilibrium characteristics and the fundamental mechanisms driving the interaction between the dyes and the surface of the modified cellulose material. The novelty of this work lies in the design of a cellulose-based adsorbent modified with a heterocyclic coumarin-thiazole ligand, which has not been previously explored for dye removal. To the best of our knowledge, this is the first report combining such a moiety with cellulose for enhanced adsorption performance against both MB and RhB dyes.

## Experimental

### Materials and chemicals

For this study, commercial cellulose powder (Sigma Aldrich, particle size of 20 μm, purity ≥ 98%) served as the principal biomass substrate for the purpose of this study. Methylene Blue (MB), Rhodamine B (RhB), and sodium periodate (NaIO_4_, 99%) were sourced from Alfa Asear. Triethylamine (C_6_H_15_N, 99%) was supplied by Sigma-Aldrich. Additional chemicals, including hydrochloric acid (HCl, 36.5–38%), absolute ethanol (CH₃CH₂OH, 99.5%), sodium chloride (NaCl, 99%), and sodium hydroxide (NaOH, 97%), were provided by Fisher Scientific. All chemicals were employed without further purification.

### Preparation of dialdehyde cellulose (DAC) & COMC adsorbent

Initially, a quantity of 1.0 g of commercial cellulose underwent a vacuum oven treatment at a temperature of 100 °C for a period of 24 h to remove any potentially adsorbed water molecules. Subsequently, DAC was synthesized by incorporating sodium periodate (NaIO_4_) into the dried commercial cellulose at a ratio of 1:2 in suspension. The reaction mixture was stirred at 45 °C for 2 h while being shielded from light exposure. Upon completion of the reaction, fibrillated dialdehyde microfibers were generated, which were then washed with deionized water followed by vacuum filtration^37,38^.

**Fig. 1:**
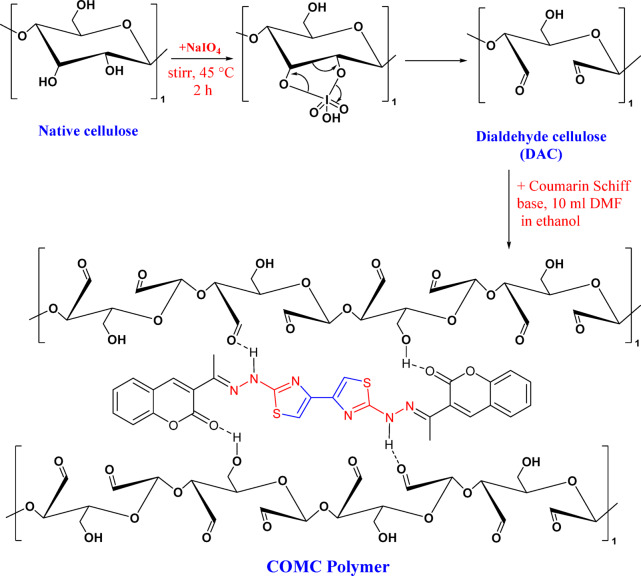
Preparation of dialdehyde cellulose (DAC) and synthesis of COMC adsorbent.

Thereafter, a suspension comprising 1.0 g of coumarin-thiazole derivatives, which had been synthesized previously^39,40^, was prepared in 10 mL of DMF and 50 mL of ethyl alcohol, and subjected to stirring within a reaction flask for 20 min. This was followed by the incorporation of 1.0 g of DAC, resulting in suspensions with a molar ratio of 1:1. The reaction was maintained for 5 h under continuous stirring at a temperature 70 °C^41^. The resultant COMC suspension underwent filtration and was washed several times with ethyl alcohol, subsequently being dried in an oven at 70 °C. The subsequent phases in the synthesis of DAC and COMC are depicted in Fig. 1.

### Material characterization

The functional groups present in both dialdehyde cellulose and the chemically modified cellulose (COMC) were identified using Fourier-transform infrared (FTIR) spectroscopy (Perkin Elmer Spectrum One, USA). Spectral data were collected across the 500–4000 cm⁻¹ wavenumber range, with a resolution of 4 cm⁻¹. Crystallographic properties were examined via wide-angle X-ray diffraction (WAXD) using a Rigaku Benchtop MiniFlex 600 diffractometer, equipped with a Cu Kα radiation source (λ = 1.5406 Å) operating at 40 kV and 15 mA. The 2θ diffraction patterns were obtained within the range of 5° and 40° at a scanning rate of 5° min^− 1^. The COMC surface morphology was analyzed using field-emission scanning electron microscopy (FESEM; ZEISS Supra 35VP, Germany) operated at 5.0 kV. Thermal stability was assessed via thermogravimetric analysis (TGA) under nitrogen atmosphere using a Shimadzu DTG-60 H analyzer, with a heating rate of 10 °C per min. The specific surface area of the COMC material was determined through Brunauer–Emmett–Teller (BET) analysis, conducted on a QUANTACHROME NOVA 2000 Series surface area instrument.

### Dye adsorption studies

The adsorption characteristics of the cationic dyes methylene blue (MB) and rhodamine B (RhB) onto the COMC material were examined via a series of controlled batch experiments. Each trial was conducted in a sealed 100 mL container, containing 50 mL of dye solution with varied amounts of the COMC adsorbent. Critical parameters such as solution pH (ranging from 2 to 9), initial dye concentration (25–250 mg/L), adsorbent dose (0.02–0.2 g), and contact time (30–300 min). All adsorption tests were performed at room temperature (25 ± 1 °C). Based on preliminary optimization, the ideal experimental conditions were determined to be 0.05 g optimal dosage of COMC, dye concentration 80 mg/L for MB and 25 mg/L for RhB, and a contact time of 120 min. The pH was modified using either 0.1 M HCl or 0.1 M NaOH. After each adsorption run, the remaining dye concentrations were measured using a UV–Vis spectrophotometer, at maximum absorbance wavelengths of 665 nm for MB and 556 nm for RhB. Dye removal efficiency (R %) and adsorption capacity at equilibrium (q_e_) were calculated according to Eqs. (1) and (2), respectively.

1 $${\text{R }}\% {\text{ }}=~\frac{{\left( {{{\text{C}}_{\text{i}}} - {{\text{C}}_{\text{e}}}} \right)}}{{{{\text{C}}_{\text{i}}}}} \times 100$$

2 $${{\text{q}}_{\text{e}}}=\frac{{\left( {{{\text{C}}_{\text{i}}} - {{\text{C}}_{\text{e}}}} \right){\text{V}}}}{{\text{m}}}$$

In this context, C_i_ and C_e_ refer to the dye concentrations at the initial and equilibrium stages, respectively, expressed in mg/L. The variable m in (g) represents the mass of the COMC adsorbent used, while V in (L) denotes the total volume of the dye solution involved in the adsorption process.

**Fig. 2:**
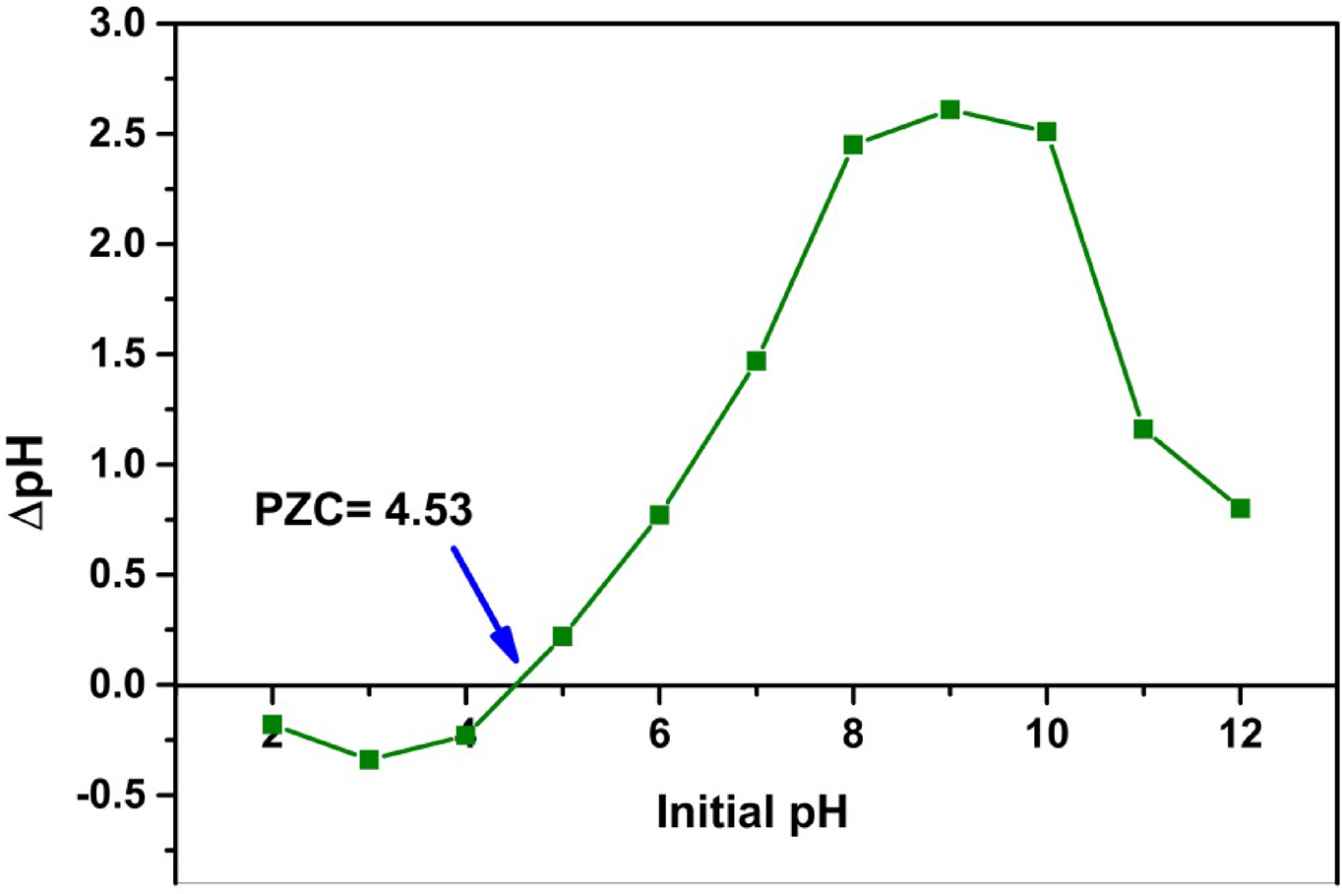
Point of zero charge of COMC adsorbent.

The point of zero charge (pH_pzc_) of the COMC adsorbent was identified using the solid addition method, as previously outlined in the literature^42^. In this method, 50 mL of a 0.1 M NaCl aqueous solution was dispensed into a series of Erlenmeyer flasks. The initial pH values (pH_i_) were adjusted across a range from 2 to 12 by incrementally adding 0.1 M HCl or 0.1 M NaOH. Afterward, 0.05 g of COMC adsorbent was introduced into each flask. The resulting mixtures were stirred at 150 rpm using a temperature-controlled shaker and maintained under room temperature conditions for 48 h to reach equilibrium. Final pH values (pH_f_) were then recorded. The pH_pzc_ was determined by plotting pH_f_ versus pH_i_ and the point of intersection with the line y = x indicated the pH_pzc_. Figure 2 graphically presents the relationship between ΔpH (defined as pH_i_ – pH_f_) and the initial pH, illustrating the surface charge behavior of the COMC adsorbent.

The adsorption mechanism of methylene blue (MB) and rhodamine B (RhB) onto the COMC adsorbent can be primarily attributed to electrostatic interactions, π–π stacking, and hydrogen bonding. Given that the pH_pzc_ of the COMC material was found to be 4.53, the surface of the adsorbent is negatively charged at solution pH values higher than the pH_pzc_. Under these conditions, strong electrostatic attraction occurs between the negatively charged adsorbent surface and the cationic dye molecules, enhancing adsorption efficiency.

Furthermore, the coumarin-thiazole functional groups introduced onto the cellulose backbone contain π-electron-rich aromatic rings, which can facilitate π–π stacking interactions with the aromatic structures of MB and RhB. Additionally, the presence of hydroxyl and nitrogen-containing groups on the COMC surface allows for potential hydrogen bonding with functional groups in the dye molecules. Collectively, these interactions contribute to the efficient adsorption performance observed. This proposed mechanism is consistent with the experimental findings.

### Adsorption isotherms

Adsorption isotherms characterize how the amount of adsorbate retained on the adsorbent surface (q_e_) varies with its equilibrium concentration in the solution, under constant temperature conditions. In this study, both the Langmuir and Freundlich isotherm models were employed in their linearized forms to analyze the adsorption behaviour and to estimate key parameters such as maximum adsorption capacity and adsorption affinity. These models are mathematically represented in Eqs. (3) and (4), respectively. One important parameter derived from the Langmuir model is the dimensionless separation factor (R_L_), which provides insight into the nature and feasibility of the adsorption process. The R_L_ parameter provides insight into the nature of the adsorption process. An R_L_ >1 indicates that adsorption is unfavourable, while R_L_=1 denotes a linear adsorption behaviour. Values between 0 and 1 signify a favourable process and an R_L_ =0 corresponds to an irreversible adsorption. This parameter serves as a critical tool in evaluating the interaction strength between the adsorbent and the adsorbate^43,44^.

3 $${{\text{C}}_{\text{e}}}/{{\text{q}}_{\text{e}}}={\text{ }}\left( {\left( {{\text{1}}/{{\text{K}}_{\text{L}}}{{\text{q}}_{{\text{max}}}}} \right){\text{ }}+{\text{ }}\left( {{{\text{C}}_{\text{e}}}/{{\text{q}}_{{\text{max}}}}} \right)} \right)$$

4 $${\text{log}}{{\text{q}}_{\text{e}}}={\text{ log}}{{\text{K}}_{\text{F}}}+{\text{1}}/{\text{n }}\left( {{\text{log}}{{\text{C}}_{\text{e}}}} \right)$$

In adsorption studies, the parameters 1/n, C_e_ (mg/L), K_F_ (mg/g), and K_L_ (L/mg), correspond to the heterogeneity index, equilibrium dye concentration, and the Freundlich and Langmuir constants, respectively. The value of 1/n serves as an indicator of adsorption favourability; values ranging from 0.1 to 0.5 denote highly favourable adsorption, whereas values greater than 2 suggest unfavourable adsorption behaviour^38^. Additionally, q_max_ and q_e_, both expressed in mg/g, correspond to the theoretical maximum adsorption capacity and the equilibrium uptake of dye, respectively.

**Fig. 3:**
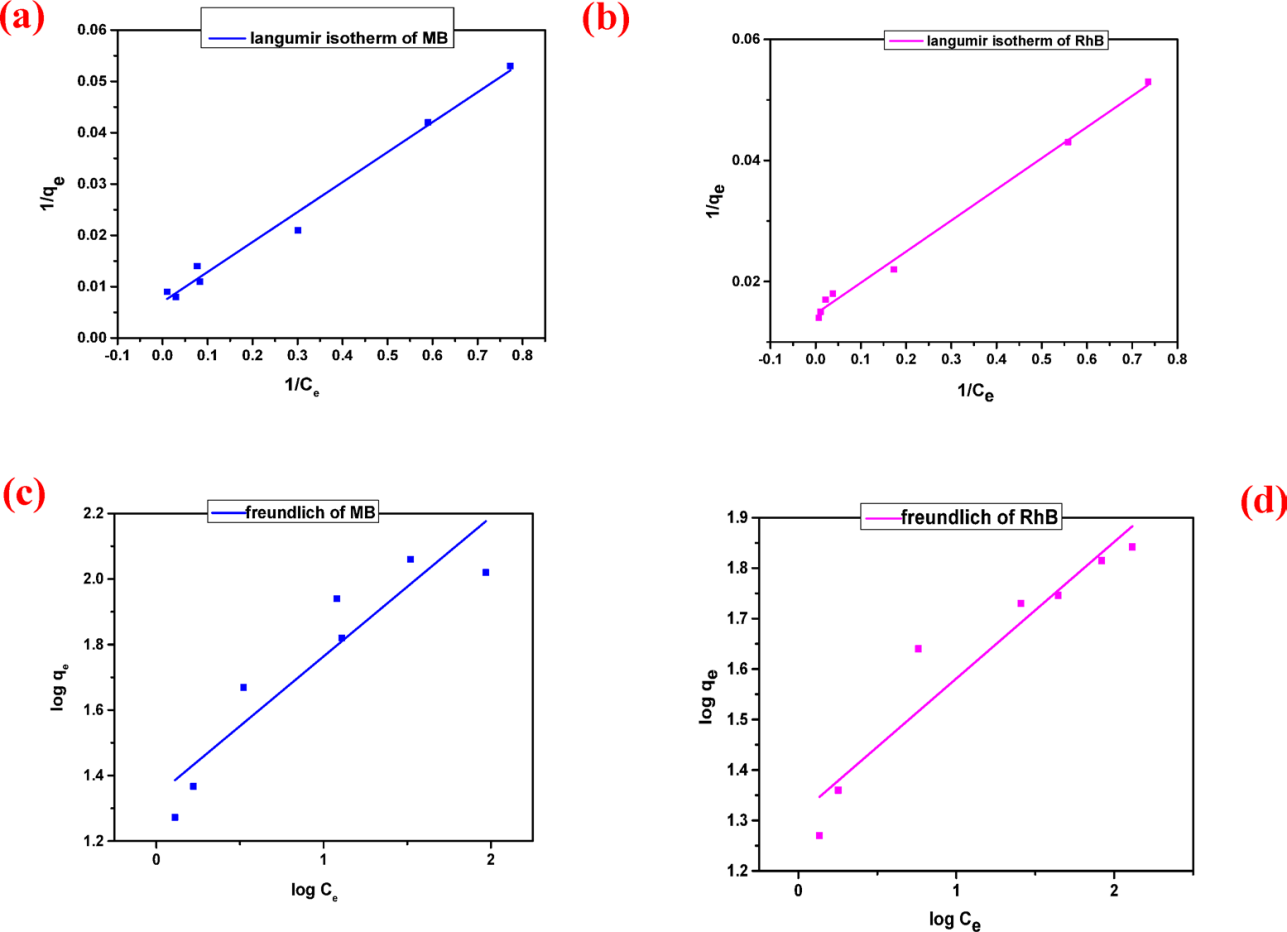
Adsorption isotherm of dyes (**a**) Langmuir model of MB, (**b**) Langmuir model of RhB, (**c**) Freundlich model of MB, and (**d**) Freundlich model of RhB adsorption by COMC adsorbent.

The suitability of the Langmuir and Freundlich isotherm models for describing the adsorption behaviour of cationic dyes on the COMC surface is illustrated in (Fig. 3). The corresponding isotherm constants K_f_, K_L_, 1/n, and q_max_ are summarized in Table 1, providing a comparative evaluation of model fitting and adsorption efficiency.

**Table 1:** Isotherm parameters for the adsorption of MB, and RhB dyes by COMC sorbent.

Dye	Langmuir parameters				Freundlich parameters		
	q_max_(mg/g)	K_L_(L/mg)	*R*_L_	*R* ^2^	K_F_	1/*n*	*R* ^2^
MB	142.247	0.120	0.094	0.985	21.802	0.425	0.838
RhB	68.49	0.284	0.123	0.993	20.44	0.270	0.901

### **Adsorption kinetics studies**

To assess the rate-controlling mechanisms involved in the adsorption of cationic dyes onto the COMC adsorbent, kinetic modelling was performed using two commonly employed approaches: the pseudo-first-order and pseudo-second-order models. These models are mathematically described by Eqs. (5) and (6), respectively. These models were employed to evaluate the adsorption kinetics and to determine the mechanism governing the interaction between the dye molecules and the adsorbent surface.

5 $${\text{ln }}\left( {{{\text{q}}_{\text{e}}} - {{\text{q}}_{\text{t}}}} \right)\,=\,{\text{ln }}{{\text{q}}_{\text{e}}}--{\text{ }}{{\text{K}}_{\text{1}}}{\text{t}}$$

6 $${\text{t}}/{{\text{q}}_{\text{t}}}={\text{ 1}}/{{\text{k}}_{\text{2}}}{{\text{q}}_{\text{e}}}^{{\text{2}}}\,+\,{\text{1}}/{{\text{q}}_{\text{e}}}$$

Within the kinetic models, K_1_ (min^–1^) and K_2_ (g mg^–1^ min^–1^) represent the rate constants for the pseudo-first-order and pseudo-second-order adsorption reactions, respectively. The term q_e_ (mg/g) indicates the adsorption capacity at equilibrium, while q_t_ (mg/g) represents the quantity of dye adsorbed at a given time t (min).

**Fig. 4:**
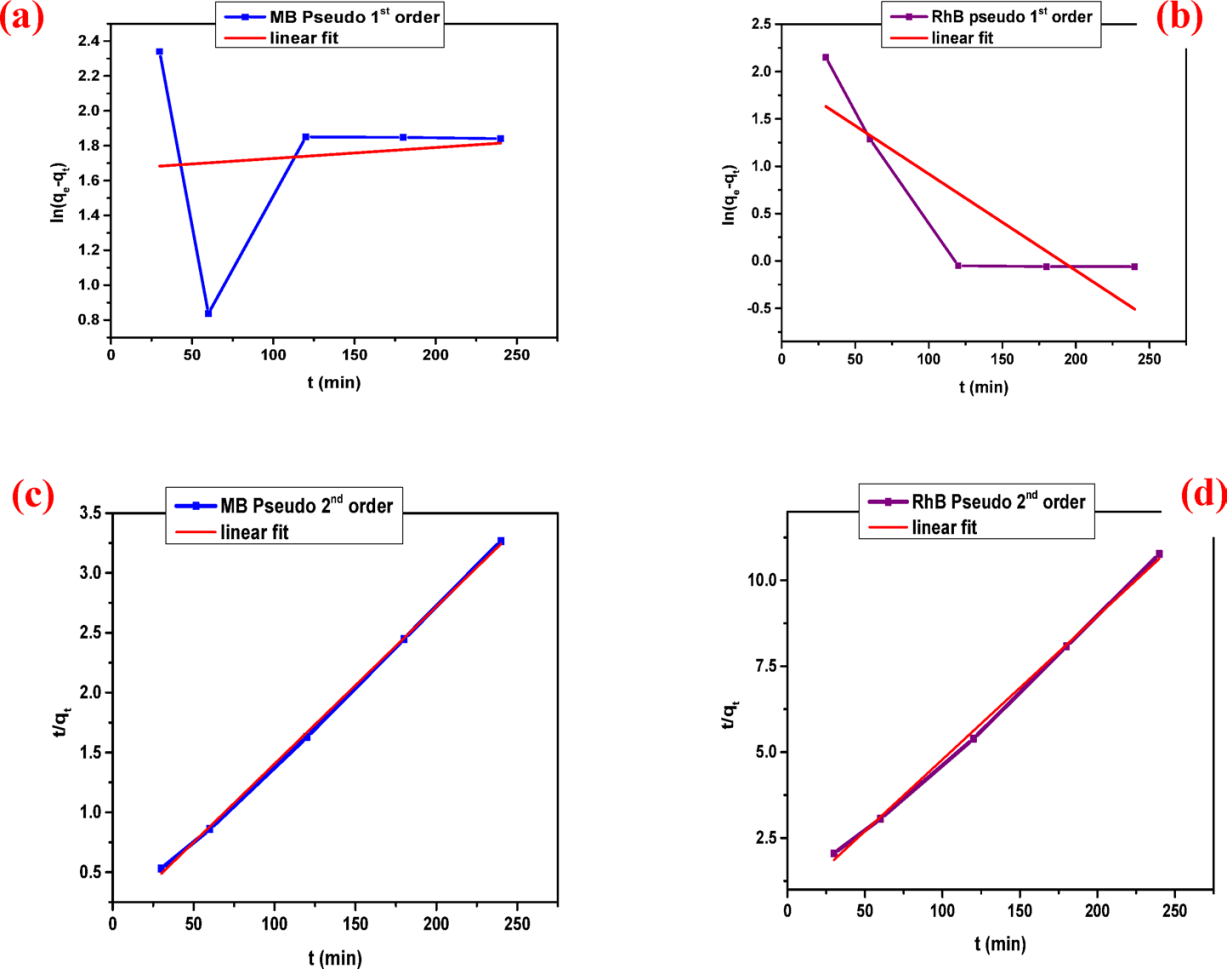
Adsorption kinetics studies of cationic dyes (**a**) Pseudo 1st order for MB adsorption, (**b**) Pseudo 1st order for RhB adsorption, (**c**) Pseudo 2nd for MB adsorption, and (**d**) Pseudo 2nd order for RhB adsorption on COMC sorbent.

As illustrated in Fig. 4, the experimental data align well with both the pseudo-first-order (PFO) and pseudo-second-order (PSO) kinetic models. The corresponding kinetic parameters including (K_1_, K_2_, q_e, exp_, q_e, cal_, and R^2^) are shown in Table 2. The adsorption of methylene blue (MB) and rhodamine B (RhB) by the COMC adsorbent reached equilibrium after 120 min. Analysis of the correlation coefficients (R²) from both kinetic models indicates that the PSO model provides a better fit for the experimental data, as shown in Table 2. This suggests that the adsorption mechanism is predominantly controlled by chemisorption, involving valence interactions through electron sharing or exchange between dye molecules and active sites on the COMC surface. Notably, the initial phase of the adsorption process (first 120 min) exhibited a rapid uptake rate, attributed to the high availability of active binding sites. As time progressed, these sites became increasingly occupied, and dye diffusion into the internal pore structure became more complex, resulting in a gradual decline in the adsorption rate.

**Table 2:** Kinetic parameters for the adsorption of MB, and RhB on COMC sorbent.

Dye	First-order model				Second-order model		
	q_e exp_. (mg/g)	q_e_,_cal_. (mg/g)	K_1_(min^− 1^)	*R* ^2^	K_2_	q_e_,_cal_(mg/g)	*R* ^2^
MB	67.13	5.279	0.052	-0.320	0.18 × 10^− 2^	76.21	0.998
RhB	23.21	6.939	-0.849 × 10^− 4^	0.652	0.28 × 10^− 2^	23.94	0.997

## Results and discussion

### Characterization

**Fig. 5:**
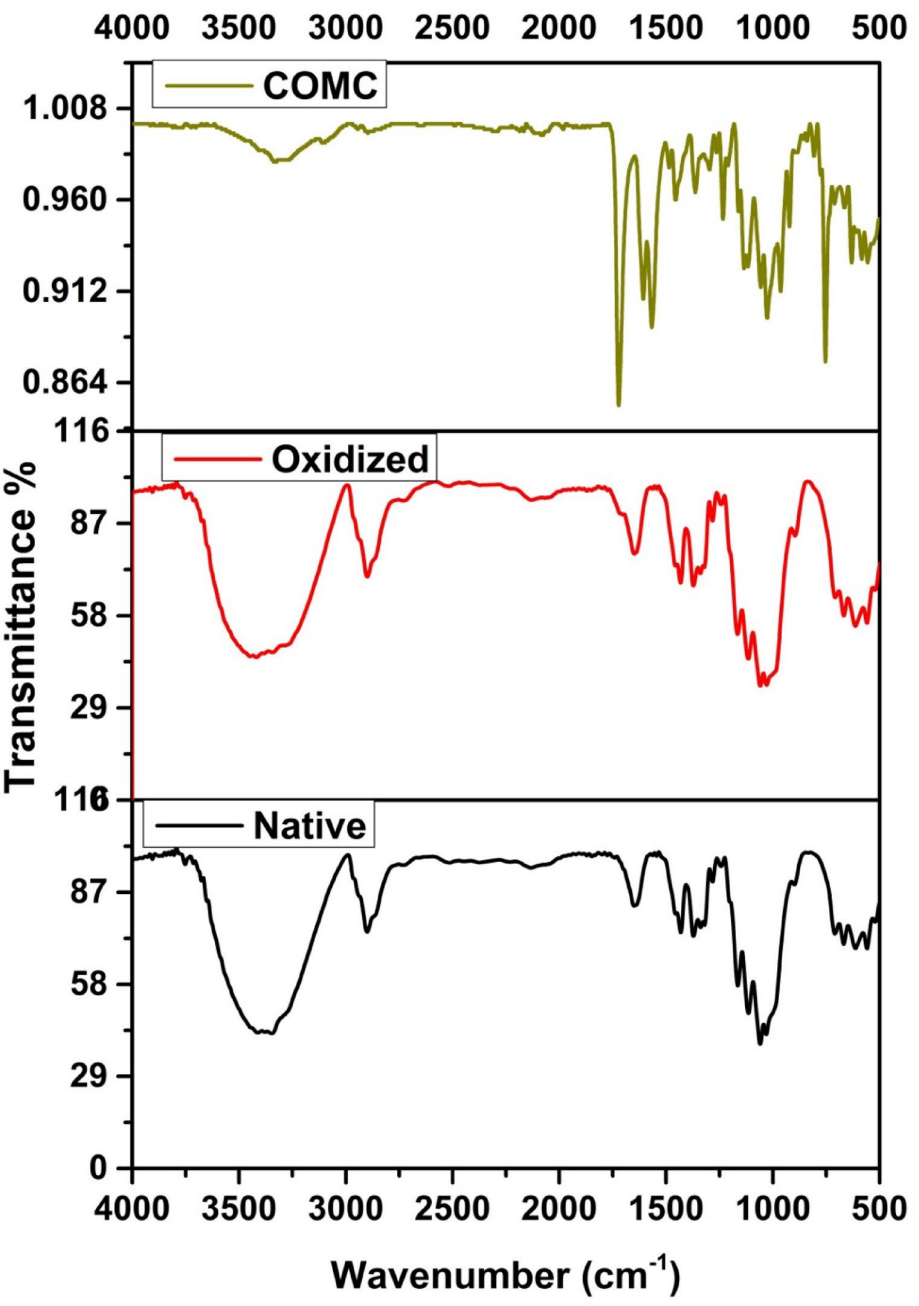
FTIR spectra of native cellulose, oxidized cellulose and COMC adsorbent.

Figure 5 presents the FTIR spectra of native cellulose fibers alongside those of its derivatives dialdehyde cellulose and coumarin-modified cellulose (COMC). The characteristic C–O stretching vibrations appear in the range of 1064 to 1189 cm^− 1^, with the native cellulose exhibiting multiple distinct absorption peaks. The OH bending and stretching vibrations are observed between 1261 and 1404 cm^–1^ and within the 3266 to 3500 cm^− 1^ region, respectively^45,46^. Moreover, the absorption bands in the range of 2794 to 2998 cm^− 1^ correspond to C–H stretching vibrations. The emergence of a new peak at 1729 cm^− 1^ in the oxidized cellulose spectrum confirms the successful periodate oxidation, attributed to the C = O stretching vibrations of the introduced aldehyde groups^38,^. In the COMC spectrum, strong absorption bands at 1719 cm^− 1^ and 1604 cm^− 1^ are assigned to C = O and C = N stretching vibrations, respectively, indicating the successful grafting of c^47^oumarin onto the oxidized cellulose backbone^40^.

**Fig. 6:**
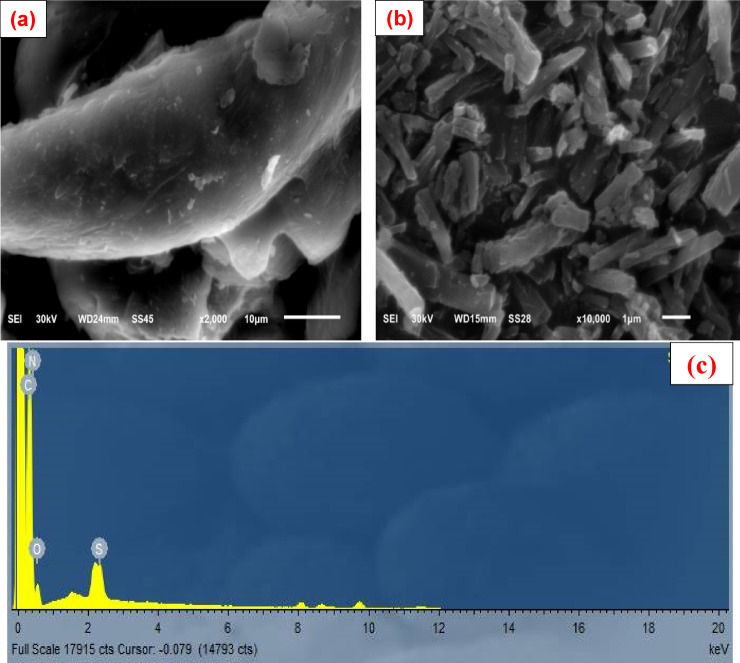
SEM images of (**a**) native cellulose, (**b**) COMC, and EDX analysis of (**c**) COMC adsorbent.

Scanning electron microscopy (SEM) was employed to observe and compare the surface morphology of the unmodified cellulose and the polymer-functionalized COMC material, as presented in Fig. 6. In contrast to the native cellulose fibres, the surface of COMC appeared notably rougher and more irregular in texture. These findings can be attributed to structural changes that occur within the fiber due to chemical interactions with coumarin-thiazole. Therefore, it is reasonable to propose the possible formation of a polymeric COMC. To validate the treatment of cellulose fibres with coumarin-thiazole derivatives, electron dispersive X-ray spectroscopy (EDX) was employed, with the results presented in (Fig. 6c). The detection of nitrogen and sulfur peaks corroborates the presence of the coumarin-thiazole compound on the cellulosic fibre.

**Fig. 7:**
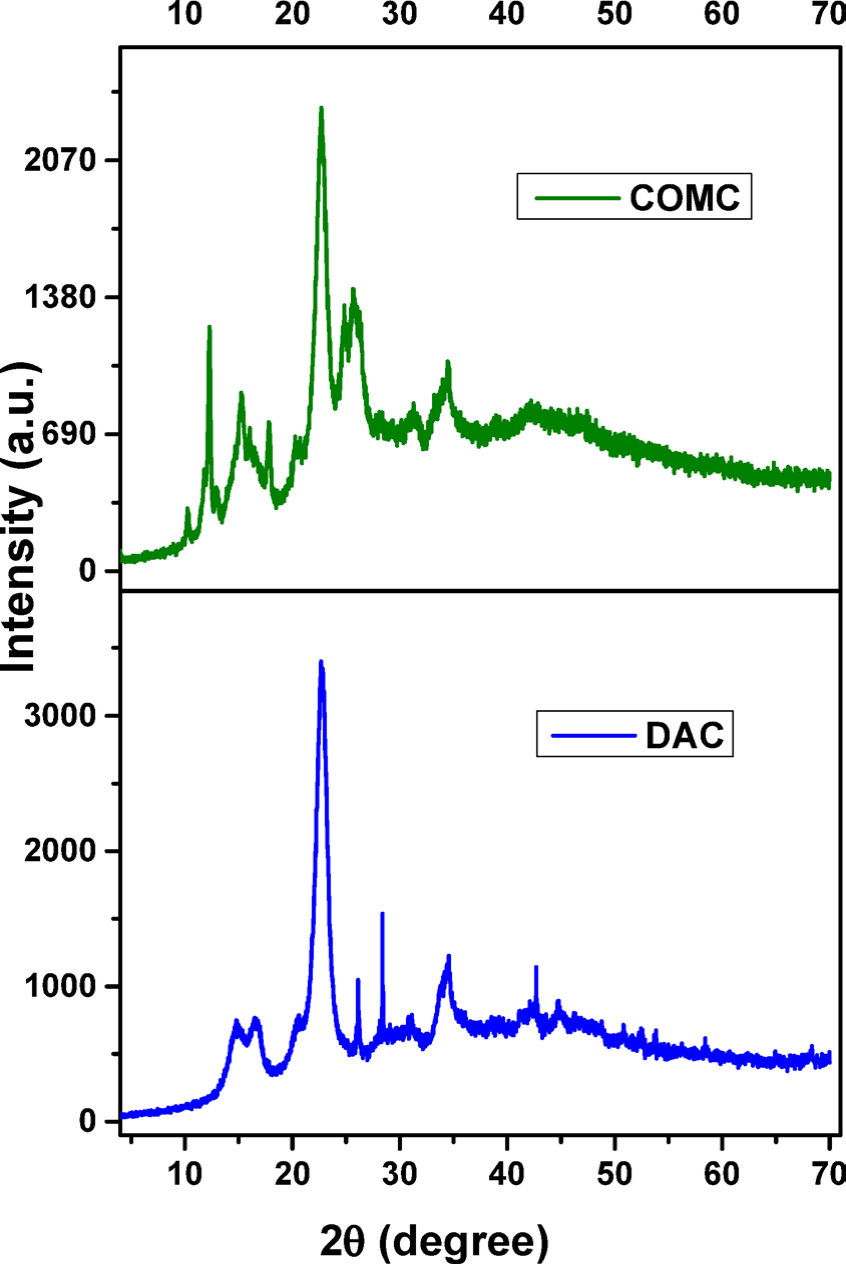
XRD pattern of DAC and COMC adsorbent.

Figure 7 displays the X-ray diffraction (XRD) patterns that show DAC and COMC. These patterns show the prominent DAC peak at 22.69°, which corresponds to the (200) reflection. Furthermore, (11̅0), (110) and (004) reflections are characterized by less intense peaks that arise at approximately 14.71°, 16.71°, and 34.57°, respectively^48,49^. Because of the amorphous portion of the prepared modified cellulose fibre, the peak at about 2θ may indicate a decrease in crystallinity^50,51^. This could suggest that the chosen chemical modification had a minor impact on the crystallinity of the functionalized samples COMC. The Segal method was used to determine the materials’ crystallinity index (CrI)^52,53^

$${\text{CrI }}=\frac{{I200~ - ~Iam}}{{I200}}~ \times {\text{1}}00$$

Where I_*am*_ is the intensity at the minimum at 2θ = 14.71° and I_*200*_ is the intensity of the crystal peak at the maximum at 2θ = 22.69°. DAC fibres have a crystallinity index of 78.66%, while COMC fibres have an index of 46.75%. COMC is more amorphous than DAC, as evidenced by the fact that its (CrI) value is lower. The denser structural appearance of COMC, relative to dialdehyde cellulose (DAC), is likely a result of variations in their underlying chemical configurations, which substantiate this observation^54^.

**Fig. 8:**
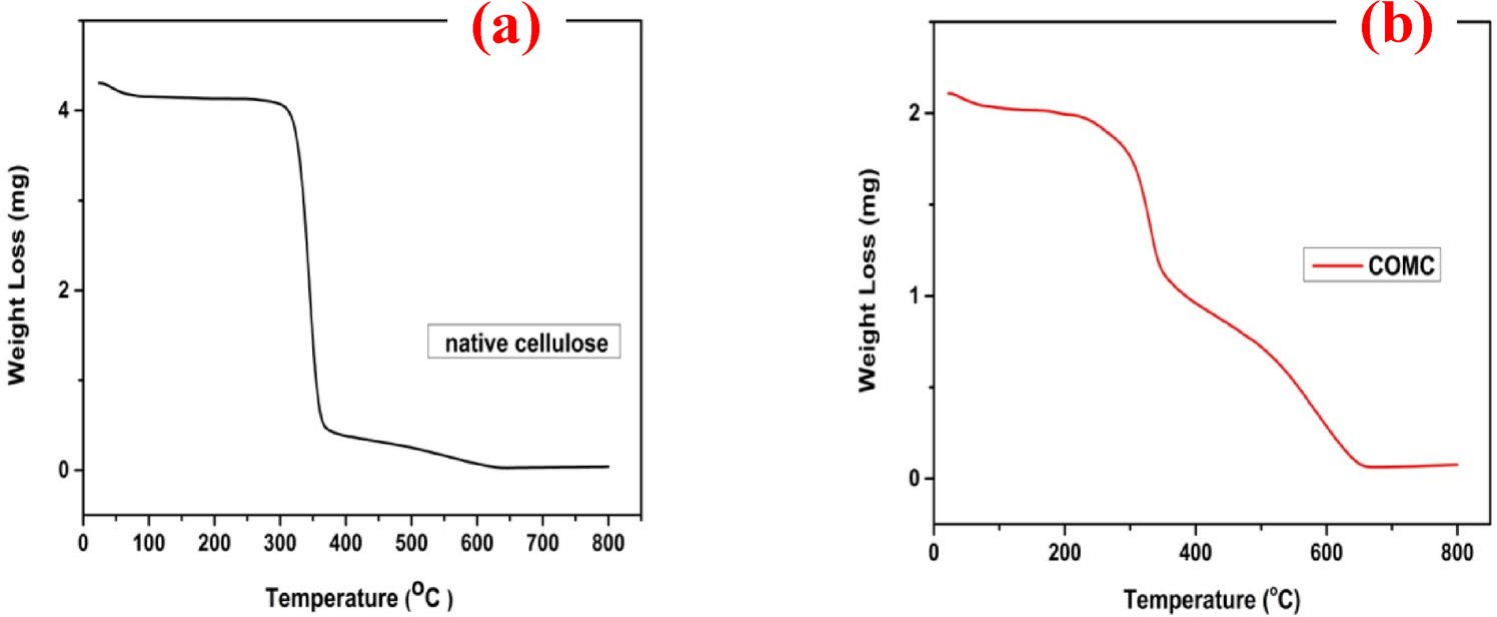
Thermal analysis (TGA) curves of (**a**) unmodified cellulose, and (**b**) of COMC adsorbent.

To provide information about the thermal stability of materials, thermogravimetric analyses were performed for both native cellulose and the adsorbent COMC at temperatures ranging from 20 to 800 °C. As illustrated in Fig. 8, each chemical component undergoes several stages of thermal degradation, as confirmed by the thermogravimetric analysis. The thermal decomposition behaviour of cellulose and COMC was investigated under a nitrogen atmosphere with a constant flow rate of 20 mL/min. The thermogram of native cellulose reveals a two-step decomposition process, typically associated with the formation of levoglucosan and anhydrocellulose^55^. Prior to chemical modification, the material retained 11.03% of its mass at 370 °C and only 0.84% at 800 °C. In contrast, the COMC adsorbent exhibited significantly higher thermal stability, with residual masses of 51.23% at 360 °C and 3.60% at 800 °C. Notably, after modification, the decomposition rate of COMC slowed between 670 °C and 800 °C. The increased final residue at 800 °C indicates enhanced thermal stability of the COMC material compared to unmodified cellulose, even under high-temperature conditions^38,56^.

**Fig. 9:**
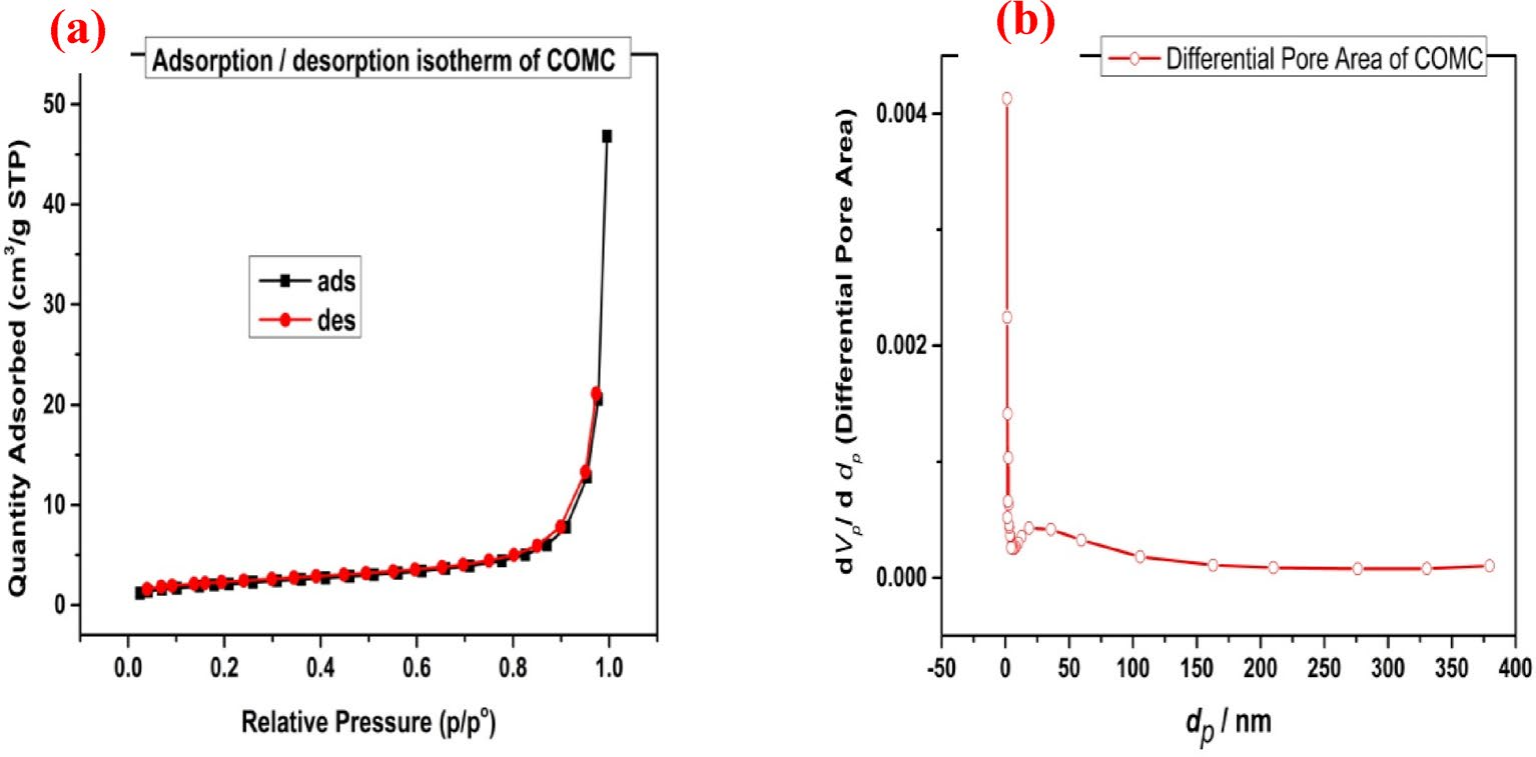
Nitrogen gas adsorption/desorption isotherm (**a**) of COMC adsorbent, and (**b**) differential pore area of COMC.

The Brunauer–Emmett–Teller (BET) nitrogen adsorption technique was employed at − 195.8 °C to evaluate key physical properties of COMC, including its specific surface area, mean pore diameter, and total pore volume. Before measurement, the sample was degassed to eliminate any residual contaminants. During analysis, the material was placed in a vacuum chamber at − 195.8 °C and exposed to varying pressure conditions. The BET analysis determined that COMC exhibited a surface area of 7.933 m²/g, a total pore volume of 0.05976 cm³/g, and an average pore diameter of 25.207 nm. Based on IUPAC classification, the resulting adsorption isotherm corresponds to type II, signifying strong interactions between the adsorbent and the adsorbate. Additionally, the nitrogen adsorption–desorption isotherms confirmed the mesoporous nature of COMC, as illustrated in Fig. 9. When compared to other common adsorbents such as paper industry sludge (0.16 m²/g), bottom ash (0.087 m²/g), and deoiled soybean residue (0.072 m²/g), COMC demonstrates a significantly higher surface area, suggesting its potential suitability for adsorption applications^57,58^.

### Adsorption study

#### Point of zero charge (pH_pzc_)

According to the calculations, the pH_pzc_ for COMC was 4.53. According to this observation, the surface of COMC exhibits a negative charge when the pH is higher than 4.53. Based on the point of zero charge pH_pzc_ for COMC cationic dyes were expected to interact more efficiently due to electrostatic attraction. A preliminary adsorption screening was performed using a set of cationic dyes, among which methylene blue (MB) and rhodamine B (RhB) showed the highest adsorption efficiency. Therefore, these dyes were selected for investigation.

#### pH influence

**Fig. 10:**
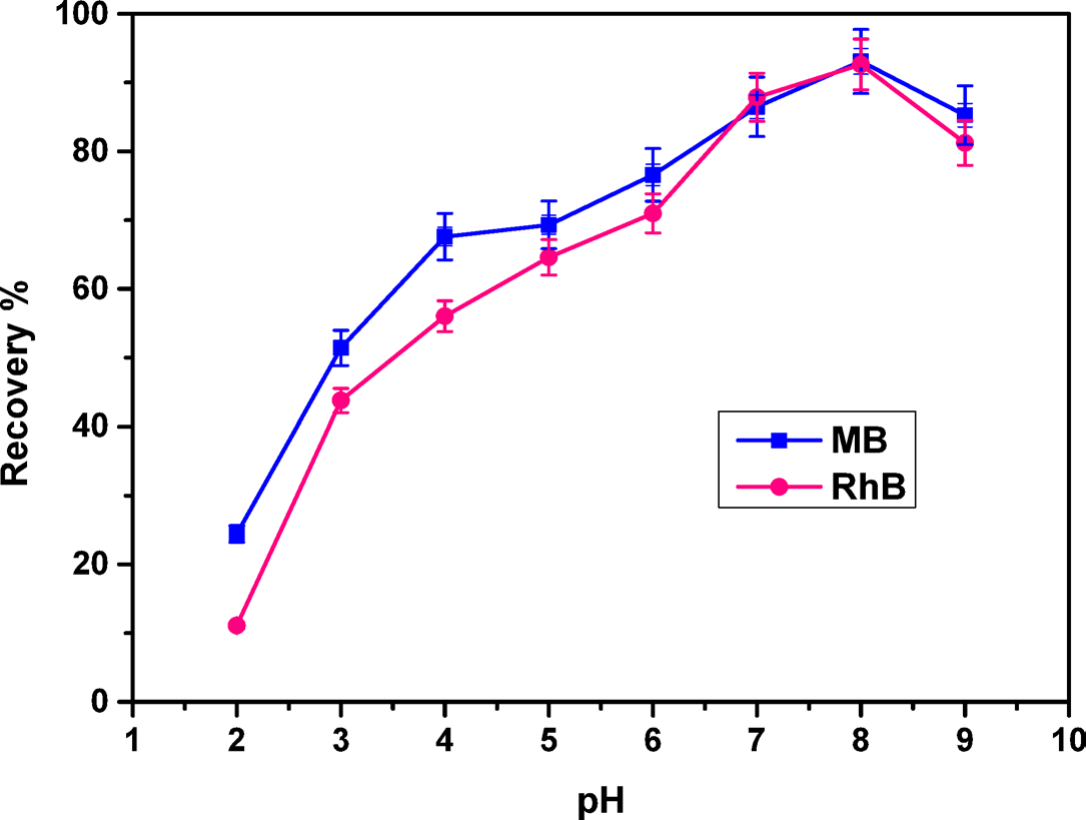
pH effect on MB and RhB adsorption onto COMC adsorbent.

The influence of pH is considered a crucial element as it affects both the solubility rate and the extent of the ionization process of the adsorbent under investigation^23,52,53^. Additionally, it may impact the speciation of dyes; the adsorption characteristics of the COMC adsorbent were evaluated within a pH range of 2 to 9. The experimental setup involved 0.05 g of the COMC compound being immersed in 50 mL of a 25 ppm RhB solution and an 80 ppm MB solution, agitated for 4 h at a temperature of 25 ℃. As shown in Fig. 10, dye adsorption increased with rising pH levels, reaching a maximum at pH 8 for both methylene blue (MB) and rhodamine B (RhB). Beyond this point, a further increase in pH resulted in a decline in adsorption efficiency. This suggests that the strong electrostatic attraction between the cationic dyes and the COMC adsorbent was the primary driver of the adsorption process within the pH range of 2 − 9, achieving an efficiency percentage of 93.08% for MB dye and 92.64% for RhB at pH 8.0. As the pH value increased, the COMC compound underwent a charge reversal, leading to a significant reduction in dye adsorption values (Fig. 10). These results suggest that electrostatic interactions are not the only mechanisms governing dye adsorption; other forces, such as hydrogen bonding and van der Waals interactions, also contribute to the process^54^. Furthermore, the data highlight the pivotal role of pH as a critical factor influencing the adsorption performance of the COMC material^43^.

#### Influence of initial concentration and adsorption isotherms

**Fig. 11:**
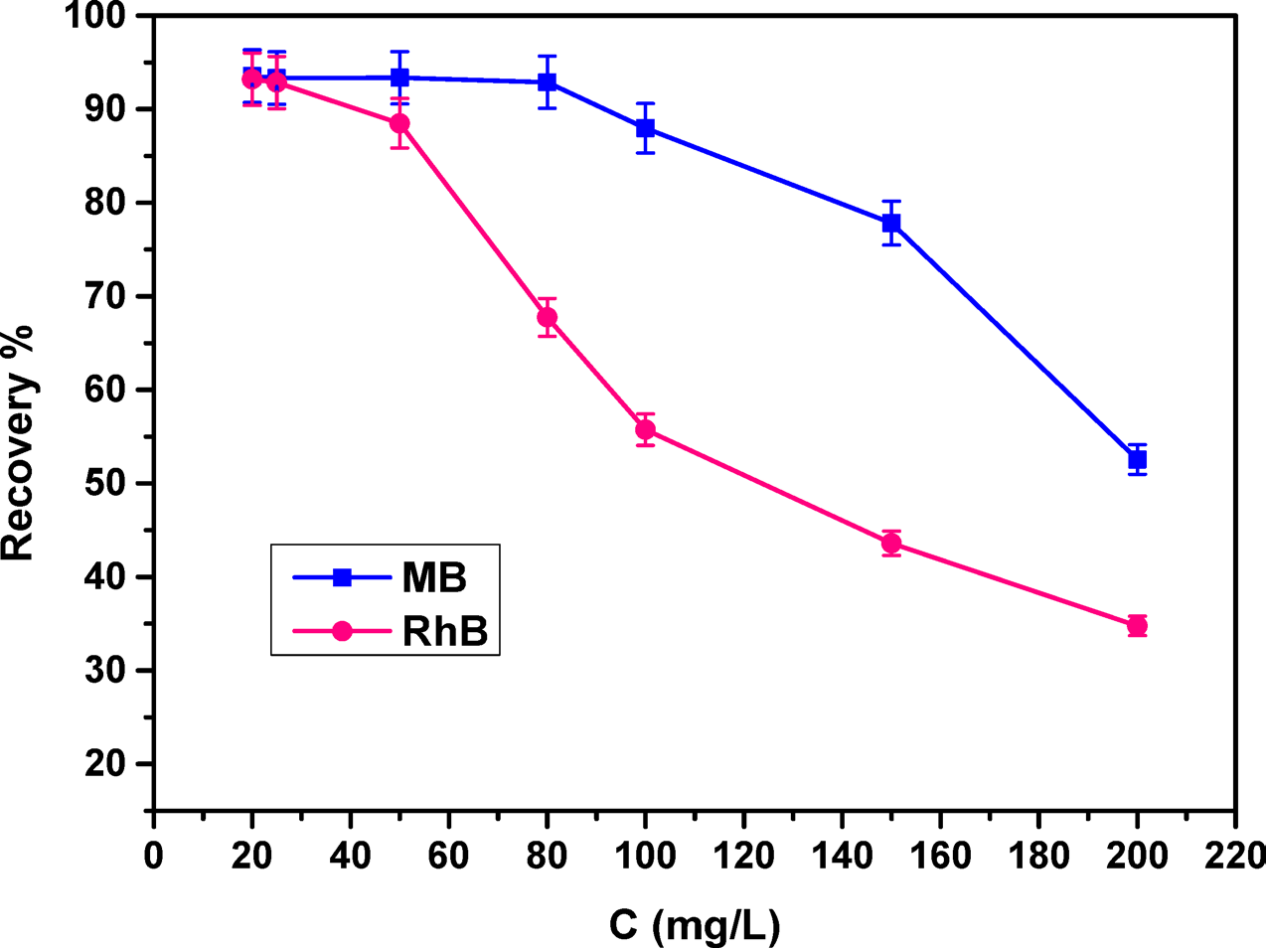
Effect of initial concentration of MB, and RhB on adsorption process.

To assess how the initial dye concentration influences the adsorption efficiency of cationic dyes, 50 mL solutions of methylene blue (MB) and rhodamine B (RhB) were prepared, each containing a fixed COMC dosage of 0.05 g. The adsorption experiments were performed at a constant pH of 8.0, with continuous agitation for 4 h at ambient temperature, while varying the initial dye concentrations within the range of 20 to 200 mg/L. The resulting adsorption efficiencies corresponding to varying initial concentrations are presented in Fig. 11. It was observed that the adsorption recovery of COMC increased with rising dye concentrations, reaching a maximum at 80 mg/L for MB and 25 mg/L for RhB. At concentrations exceeding this range, a noticeable reduction in adsorption efficiency was observed, which is likely attributed to the saturation of active binding sites on the COMC surface^57,59^.

The adsorption performance of COMC aligns more closely with the Langmuir isotherm model, as evidenced by a higher correlation coefficient (R²) relative to that of the Freundlich model, as shown in Table 1. This outcome implies that dye molecules interact with the COMC surface primarily through monolayer chemisorption, involving site-specific binding interactions. Additionally, the dimensionless separation factor R_L_, calculated and reported in Table 1, was found to lie between 0 and 1, confirming the favorability of the adsorption process. Collectively, these results support the effectiveness of COMC as a promising material for removing dyes from aqueous media.

#### Effect of adsorbent dose

**Fig. 12:**
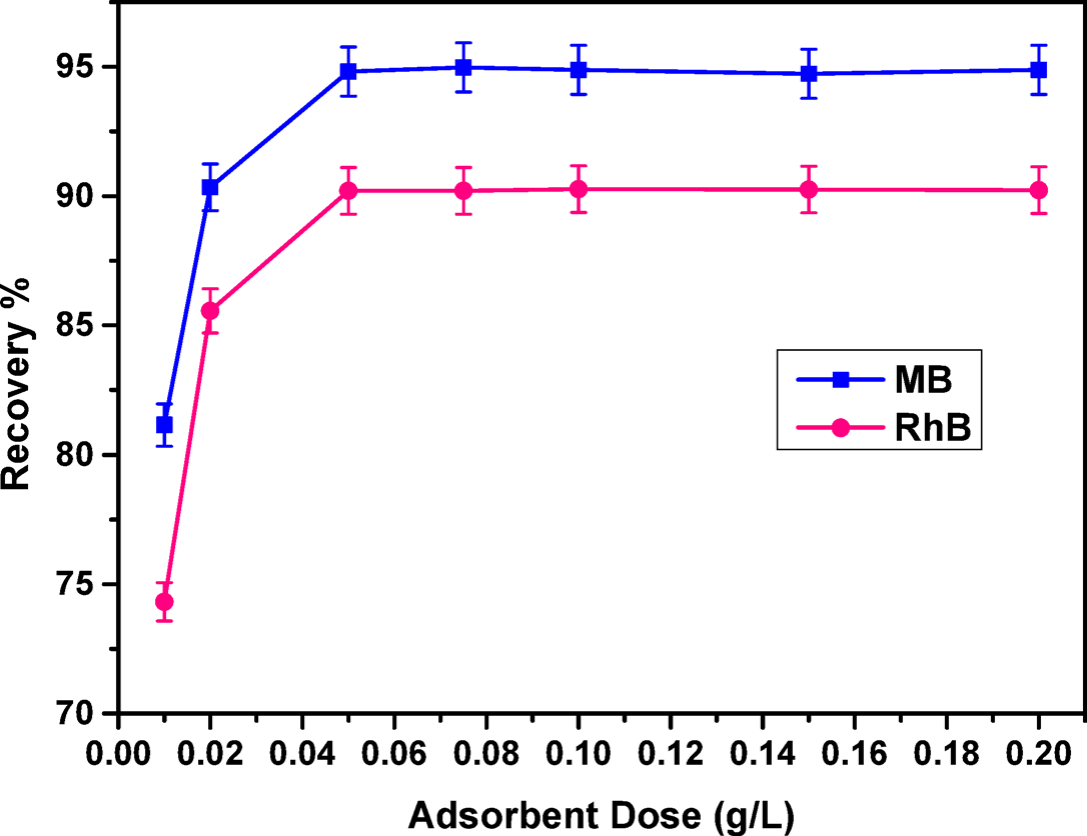
Effect of dose on MB, and RhB adsorption on COMC at room temperature for 120 min.

The effect of COMC adsorbent dosage on dye removal efficiency (%) was examined within the range of 0.01 g to 0.2 g, under fixed experimental conditions with continuous shaking for 4 h at ambient temperature. The results are illustrated in Fig. 12. A significant enhancement in the removal efficiency of both methylene blue (MB) and rhodamine B (RhB) was observed as the adsorbent dosage increased from 0.01 g to 0.05 g. However, beyond this level, further increases in COMC amount led to only marginal improvements in adsorption performance. This plateau behaviour is most likely attributed to the saturation of active binding sites on the adsorbent surface, thereby restricting additional dye adsorption^38,60^.

#### Influence of contact time

**Fig. 13:**
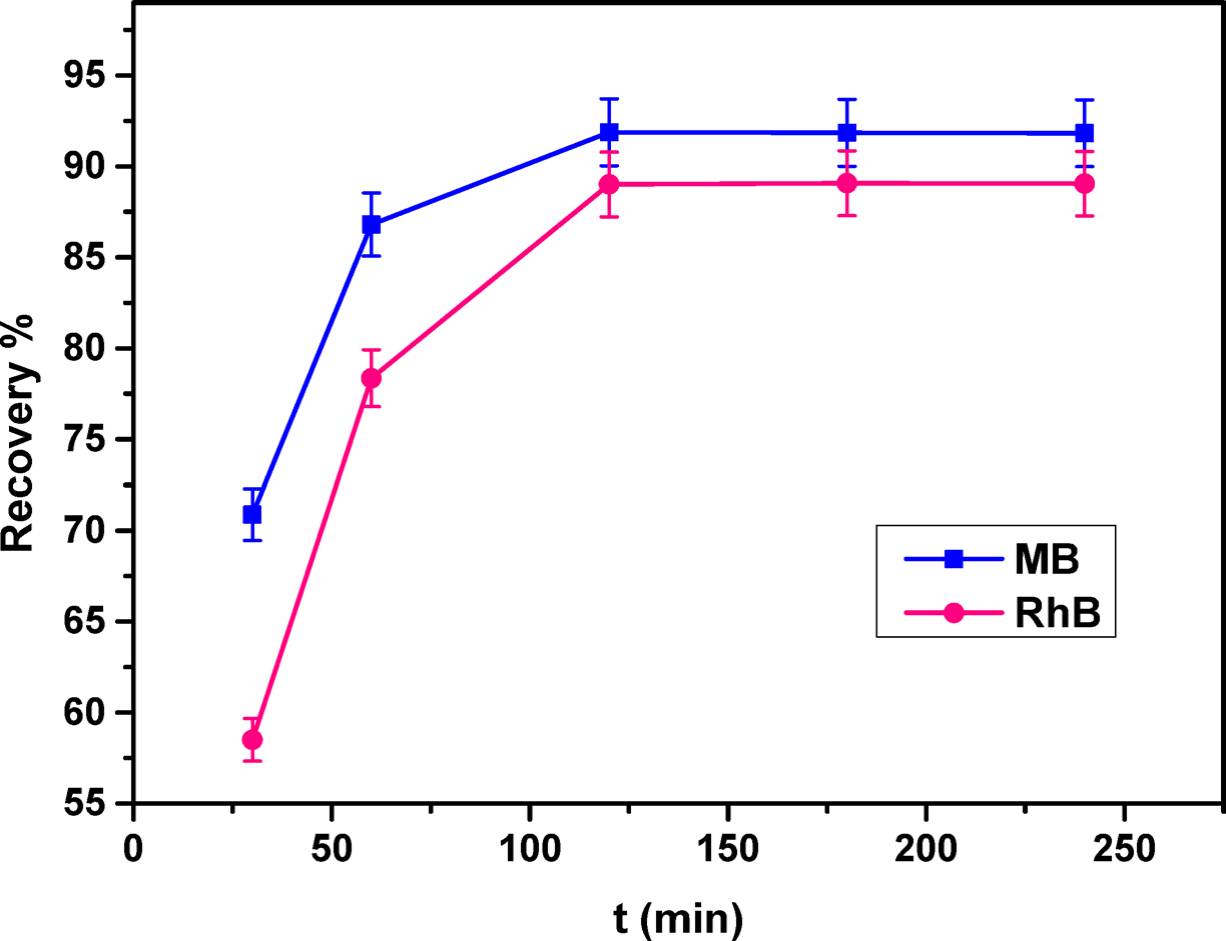
Effect of time on MB, and RhB adsorption on COMC sorbent.

To better understand the adsorption mechanisms of methylene blue (MB) and rhodamine B (RhB) on the COMC adsorbent, a series of kinetic experiments were carried out by varying the contact time from 30 to 240 min. In these trials, 0.05 g of COMC was added to glass vessels containing 50 mL dye solutions with concentrations of 80 mg/L for MB and 25 mg/L for RhB, both adjusted to pH 8.0 and kept at room temperature. The adsorption performance at different contact times is depicted in Fig. 13. The results showed that dye removal efficiency (%) increased progressively with time and stabilized within the interval of 120 to 240 min. accordingly, the equilibrium time for the adsorption of both dyes onto COMC was established at 120 min.

#### Effect of temperature and thermodynamic studies

**Fig. 14:**
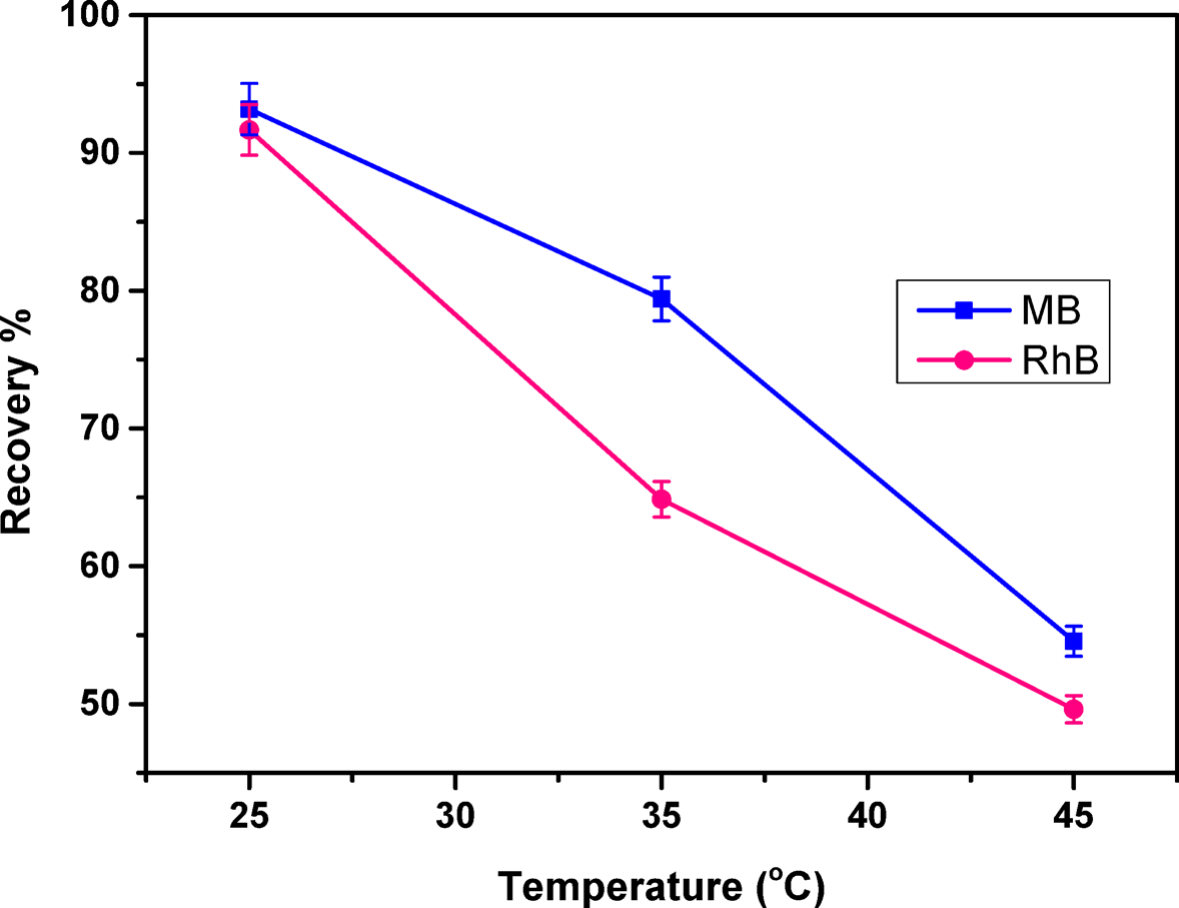
Effect of temperature on MB, and RhB adsorption on to COMC adsorbent.

To evaluate the effect of temperature on adsorption performance, equilibrium isotherms for COMC were generated across a temperature range of 298 to 318 K, as illustrated in Fig. 14. The findings revealed that adsorption capacity (%) declined with increasing temperature, suggesting that the adsorption of methylene blue (MB) and rhodamine B (RhB) onto the COMC surface is governed by an exothermic process.

**Fig. 15:**
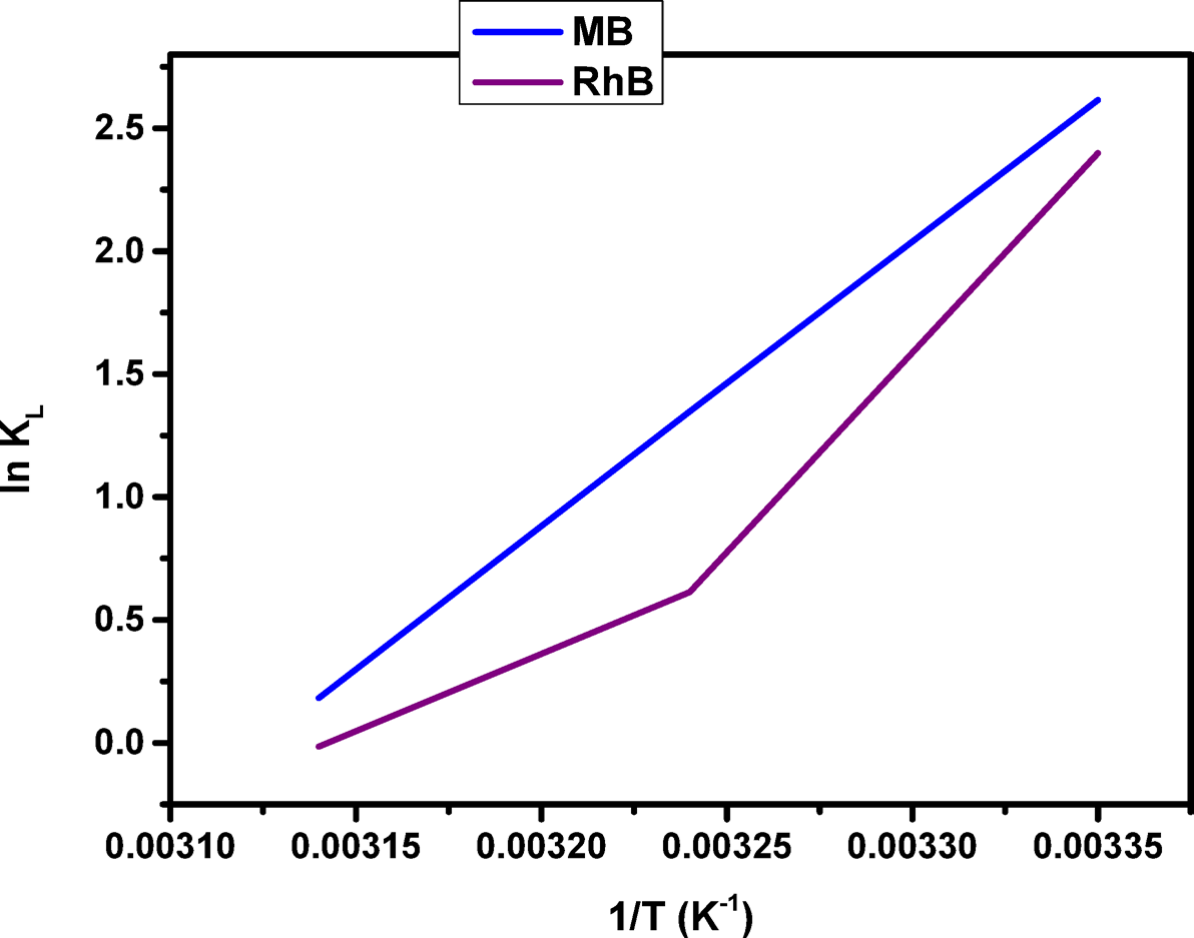
Plot of ln K_L_ versus (1/T) absolute temperature for the adsorption of MB, and RhB dye on to COMC adsorbent.

To gain deeper insight into the adsorption mechanism, the thermodynamic aspects of the process including feasibility, spontaneity, and molecular disorder at the solid–liquid interface were examined. Accordingly, the standard thermodynamic parameters, namely Gibbs free energy change (ΔG°_ads_), entropy change (ΔS°_ads_), and enthalpy change (ΔH°_ads_), were determined over the temperature range of 25 to 45 °C. These parameters provide insight into the nature of the adsorption process, where ΔG° reflects spontaneity, ΔH° indicates the heat exchange involved, and ΔS° measures the degree of disorder at the interface. The thermodynamic values were determined using Eqs. (7), (8), and (9), based on the relationship between ln K_L_ and the reciprocal of temperature (1/T) in Kelvin, as depicted in Fig. 15.

7 $${{\text{K}}_{\text{L}}}={\text{ }}{{\text{q}}_{\text{e}}}/{\text{ }}{{\text{C}}_{\text{e}}}$$

8 $$\Delta \,{{\text{G}}^{\text{o}}}={\text{ }} - {\text{RTln}}{{\text{K}}_{\text{L}}}$$

9 $$\Delta {{\text{G}}^{\text{o}}}={\text{ }}\Delta {{\text{H}}^{\text{o}}} - {\text{T}}\Delta {{\text{S}}^{\text{o}}}$$

The parameters K_L_, q_e_, and C_e_ are commonly recognized as thermodynamic constants that characterize the equilibrium state of the adsorption process. In this context, q_e_ (mg/g) corresponds to the equilibrium uptake of dye by the COMC adsorbent, while C_e_ (mg/L) represents the dye concentration remaining in solution at equilibrium. R denotes the universal gas constant. As shown in Table 3, the negative values of ∆H^o^ and ∆G^o^ confirm that the adsorption of dyes onto COMC is both exothermic and spontaneous in nature. Additionally, the negative ∆S^o^ value indicates reduced randomness at the solid–liquid interface, implying a more organized configuration of dye molecules on the surface of the adsorbent during the adsorption process.

**Table 3:** Thermodynamic parameters for the adsorptive uptakes of MB, and RhB onto COMC sorbent.

Dye	Temperature (K)	∆G (kJ/mol)	∆H (kJ/mol)	∆S (J/mol.K)
MB	298	-6.478	-96.31	-300.89
	308	-3.451		
	318	-0.481		
RhB	298	-5.943	-117.48	-371.57
	308	-1.569		
	318	0.039		

## Desorption and recyclability study

**Fig. 16:**
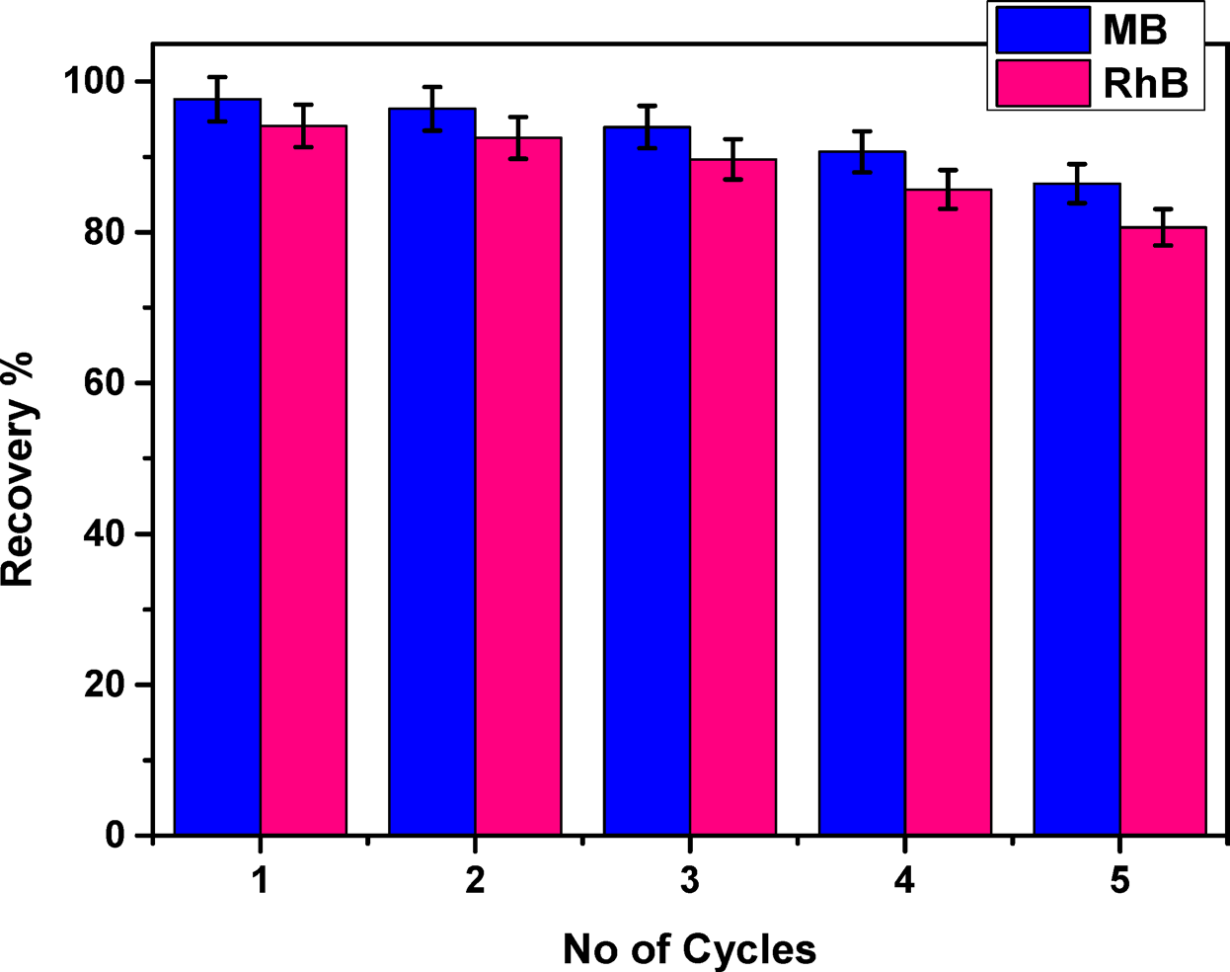
Regeneration efficiency of COMC adsorbent.

The reusability of the COMC adsorbent for removing methylene blue (MB) and rhodamine B (RhB) was assessed across five successive adsorption desorption cycles, as depicted in Fig. 16. A range of eluents was tested for desorbing the cationic dyes from the COMC surface, including ethanol, HCl (0.2 M), NaHCO_3_ (0.1 M), NaOH (0.2 M), HNO_3_ (0.1 M), and K_2_CO_3_ (0.1 M). Among these, 0.1 M HNO_3_ was found to be the most effective desorbing agent and was consequently employed for elution at ambient temperature. The desorption capacity showed a gradual decline, decreasing from 97.65% in the first cycle to 80.65% by the fifth cycle. This reduction may be attributed to irreversible interactions between dye molecules and specific adsorption sites on the COMC surface, as well as to minor losses of adsorbent material due to repeated washing and partial desorption. Despite this decline, the COMC adsorbent demonstrated substantial reusability, retaining a high level of adsorption efficiency after multiple cycles. These results highlight the effectiveness of COMC as a renewable and eco-friendly biosorbent for the removal of cationic dyes from aqueous environments, further supporting its applicability in real-world water treatment systems.

## Applications

**Fig. 17:**
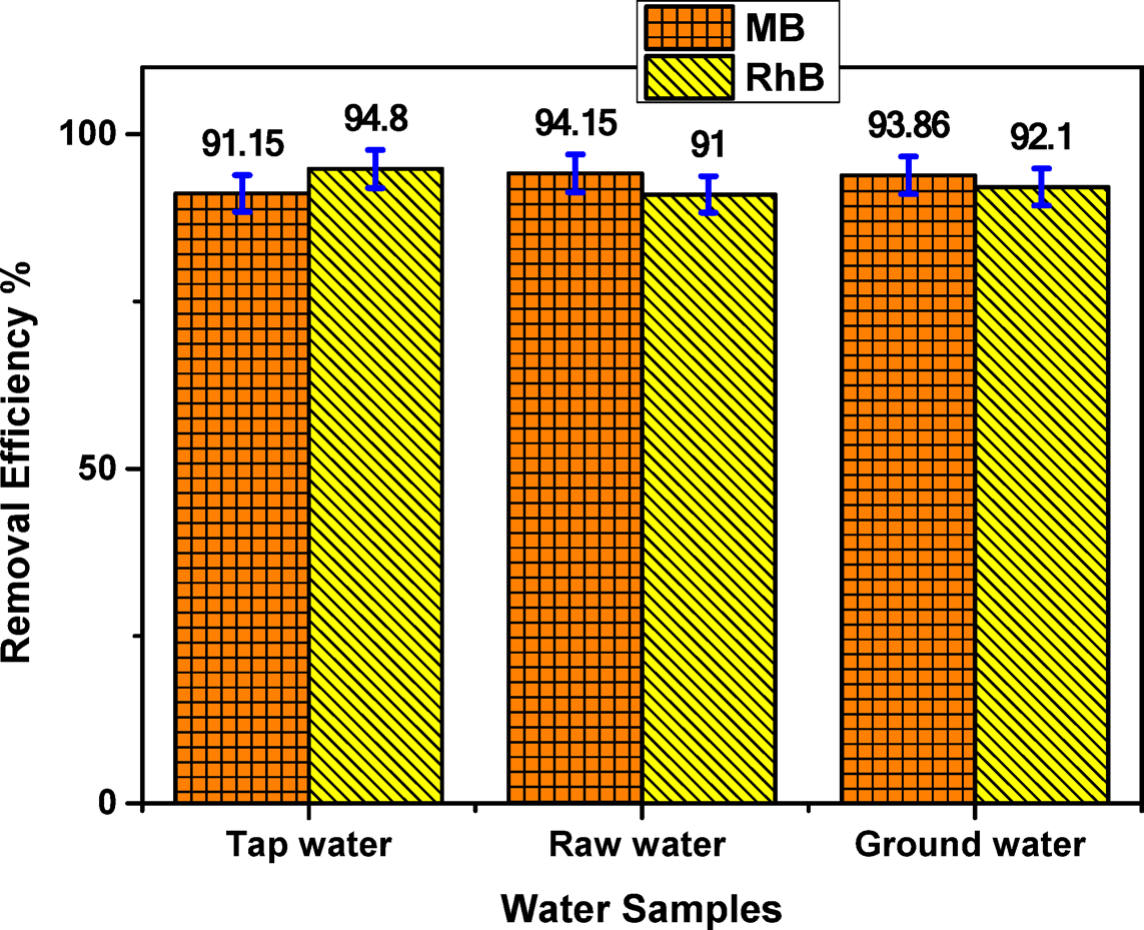
Adsorption efficiency of dye in real water samples by COMC adsorbent.

To examine the real-world applicability of the COMC adsorbent, adsorption experiments were carried out using methylene blue (MB) and rhodamine B (RhB) in various natural water samples, including tap water, untreated surface water (raw water), and groundwater. These evaluations were designed to simulate realistic environmental conditions and assess the material’s effectiveness accordingly. As illustrated in Fig. 17, the COMC adsorbent achieved removal efficiencies exceeding 91% for both dyes across all tested water sources. These results demonstrate that the COMC adsorbent retains its high adsorption capacity in complex water systems, despite the potential presence of competing ions and organic matter. The high recovery rates confirm the suitability and robustness of COMC for removing cationic dyes from real aqueous environments, highlighting its potential for practical application in wastewater treatment processes.

Although the adsorption tests were conducted using synthetic dyes spiked into real wastewater samples, the high efficiency of the developed adsorbent under a wide range of pH and dye concentrations suggests its potential for application in real industrial effluents. Future work will focus on testing the material with untreated industrial wastewater samples containing mixed pollutants to evaluate its performance in more complex matrices.

## Comparison of the proposed adsorbent COMC with other cited adsorbents

A comparative evaluation of the adsorption performance of COMC with other reported adsorbents from the literature is presented in Table 4. In evaluating various adsorbents for separation, it is essential to consider factors such as adsorption capacity, dosage, and the type of adsorbent used. As indicated in Table 4, compared to other adsorbents documented in previous studies, the COMC material exhibits notably higher adsorption capacities for the recovery of cationic dyes such as methylene blue (MB) and rhodamine B (RhB).

**Table 4:** Comparison of adsorption capacity of MB, and RhB dye onto COMC with previously reported studies.

Dye	Adsorbent	Adsorbent dose	Adsorption capacity (mg/g)	References
RhB	Tamarind fruit shell Activated carbon	0.5 g	3.943	^61^
	Corn cobs activated carbon	0.3 g	5.92	^62^
	Fly ash	1.0 g	2.33	^63^
	Natural diatomite	1.0 g	8.13	^64^
	Mimusops Elengi activated carbon	0.4 g	1.70	^65^
	Exhausted coffee ground powder	50 mg	5.26	^29^
	Paper industry waste sludge	2.0 g	6.71	^57^
	COMC	0.05 g	68.49	Present study
MB	Sponge-gourd fibers/hydroxyapatite composite	0.4 g	25	^66^
	Chitosan-montmorillonite/polyaniline	0.05 g	111	^67^
	Bi_2_O_3_-SrO-FeO@SiO_2_	20 mg	244	^68^
	CMC/PAA/GO	0.05 g	138	^69^
	GO/g-C_3_N4-Fe_3_O_4_	0.03 g	187.36	^70^
	COMC	0.05 g	142.24	Present study

## Conclusions

A chemically modified cellulosic adsorbent (COMC) was successfully synthesized and demonstrated high efficiency in the adsorption of cationic dyes from aqueous solutions. The structural and physicochemical features of COMC were thoroughly investigated using a combination of analytical methods, including SEM, EDX, XRD, FTIR, BET, pH_PZC_, and TGA analyses. The structural characterization confirmed the successful functionalization, and the material exhibited maximum adsorption capacities of 142.24 mg/g for methylene blue (MB) and 68.49 mg/g for rhodamine B (RhB). The effects of several operational parameters such as initial dye concentration, solution pH, contact time, temperature, and sorbent dosage were systematically evaluated to enhance the adsorption performance. Kinetic modelling indicated that the adsorption process follows the pseudo-second-order (PSO) model, whereas the equilibrium data conformed best to the Langmuir isotherm (R^2^ > 0. 985), pointing to monolayer adsorption behaviour on a uniform surface. Thermodynamic analysis further supported the spontaneity and exothermic nature of the adsorption, with negative values of ΔG^o^, ΔH^o^, and ΔS^o^, indicating a chemisorption mechanism. The COMC adsorbent offers several advantages, including high adsorption capacity, good thermal stability, and excellent reusability over multiple cycles. Moreover, it also maintained > 91% removal efficiency in real water matrices, highlighting its potential for practical wastewater treatment applications. Future work will focus on scaling up the synthesis, applying the material to untreated industrial effluents containing complex dye mixtures, and exploring its regeneration performance under continuous flow conditions.

## Data Availability

All data generated or analyzed during this study are included in this published article.
